# In Vitro and In Ovo Host Restriction of Aquatic Bird Bornavirus 1 in Different Avian Hosts

**DOI:** 10.3390/v12111272

**Published:** 2020-11-07

**Authors:** Alexander Leacy, Éva Nagy, Phuc H. Pham, Leonardo Susta

**Affiliations:** Department of Pathobiology, Ontario Veterinary College, University of Guelph, Guelph, ON N1G 2W1, Canada; aleacy@uoguelph.ca (A.L.); enagy@ovc.uoguelph.ca (É.N.); phpham@uoguelph.ca (P.H.P.)

**Keywords:** avian bornavirus, *Waterbird orthobornavirus 1*, ABBV-1, host restriction, growth kinetics, in ovo infection

## Abstract

Aquatic bird bornavirus 1 (ABBV-1) is associated with chronic meningoencephalitis and ganglioneuritis. Although waterfowl species act as the natural host of ABBV-1, the virus has been sporadically isolated from other avian species, showing the potential for a broad host range. To evaluate the host restriction of ABBV-1, and its potential to infect commercial poultry species, we assessed the ability of ABBV-1 to replicate in cells and embryos of different avian species. ABBV-1 replication was measured using multi- and single-step growth curves in primary embryo fibroblasts of chicken, duck, and goose. Embryonated chicken and duck eggs were infected through either the yolk sac or chorioallantoic cavity, and virus replication was assessed by immunohistochemistry and RT-qPCR in embryonic tissues harvested at two time points after infection. Multi-step growth curves showed that ABBV-1 replicated and spread in goose and duck embryo fibroblasts, establishing a population of persistently infected cells, while it was unable to do so in chicken fibroblasts. Single-step growth curves showed that cells from all three species could be infected; however, persistence was only established in goose and duck fibroblasts. In ovo inoculation yielded no detectable viral replication or lesion in tissues. Data indicate that although chicken, duck, and goose embryo fibroblasts can be infected with ABBV-1, a persistent infection is more easily established in duck and goose cells. Therefore, ABBV-1 may be able to infect chickens in vivo, albeit inefficiently. Additionally, our data indicate that an in ovo model is inadequate to investigating ABBV-1 host restriction and pathogenesis.

## 1. Introduction

Aquatic bird bornavirus 1 (ABBV-1) has been associated with chronic meningoencephalitis and ganglioneuritis in several species of migratory waterfowl (e.g., Canada geese, trumpeter swans, mute swans) throughout Canada and the United States [1,2]. ABBV-1 is a virus of the species *Waterbird 1 orthobornavirus*, in the genus *Orthobornavirus*, family *Bornaviridae* and order *Mononegavirales* [3]. Bornaviruses (i.e., viruses in the family *Bornaviridae*) have a non-segmented, negative-sense, single-stranded RNA genome, which is roughly 9 kb and contains six genes encoding five structural and one non-structural proteins: nucleoprotein (N), non-structural protein (X), phosphoprotein (P), matrix (M), glycoprotein (G), and RNA dependent RNA polymerase (large; L) [4,5].

Bornaviruses do not cause cell lysis, and rather induce a persistent infection through multiple mechanisms, including inhibition of the host immune response, negative regulation of cellular transcription by the viral X protein, and close associations with host chromosomes [6,7]. There is limited release of infectious virions during the replication cycle, and virus propagation mainly occurs by cell-to-cell spread, which is mediated by the G protein [8]. Although primarily neurotropic in vivo, bornaviruses are able to establish persistent infection in a variety of cell types in vitro, typically without production of cytopathic effect (CPE) [4,9].

Viruses in the *Psittaciform 1* and *2 orthobornavirus* species are the causative agents of proventricular dilatation disease (PDD) in psittacines (i.e., parrots and allies); a disease characterized by inflammation of the ganglia in the gastrointestinal (GI) tract, which causes impaired intestinal motility, leading to maldigestion, starvation, and ultimately death [10]. Common microscopic lesions include lymphoplasmacytic perivascular cuffs in the peripheral and central nervous system [10]. Pathological findings in ABBV-1-infected waterfowl are similar to what observed with PDD, although inflammation in the central nervous system appears to be more common compared to psittacines with PDD [11].

While the typical host range of ABBV-1 includes geese and swans, ABBV-1 has been detected in birds taxonomically distinct from waterfowl, including eagles [12], gulls [13], and an emu [14]. Histological lesions in some of these birds were consistent with what is reported in waterfowl. Similarly, other avian bornaviruses (i.e., bornaviruses isolated from birds) have been shown to have a broad host range, outside the common avian reservoirs. For instance, a Himalayan monal (*Lophophorus impejanus*; order *Galliformes*), presented with PDD-like clinical signs and post-mortem findings, tested positive for parrot bornavirus-4 (PaBV-4 genotype, in the *Psittaciform 1 orthobornavirus* species) [15].

Despite the high prevalence in waterfowl, and propensity to cause disease in multiple avian taxa, the ability of ABBV-1 to infect and cause disease in poultry species has not been evaluated. Infection and establishment of ABBV-1 in poultry is a hazard that could lead to potential economic losses. In this study, the ability of ABBV-1 to infect and replicate in cells of chicken- and waterfowl-origin was evaluated using both in vitro and in ovo models. First, replication kinetics of ABBV-1 were determined using multi- and single-step growth curves in primary embryo fibroblasts of chicken, duck, and goose. Second, embryonated eggs of chicken and duck species were inoculated into either the yolk or chorioallantoic cavity with ABBV-1, and embryos were harvested at mid or late embryonation. Lesion development in tissues was assessed by histopathology, and virus replication was evaluated by immunohistochemistry (IHC) and RT-qPCR for viral RNA.

## 2. Materials and Methods

### 2.1. Eggs and Cells

Fertilized White Leghorn chicken, Pekin duck, and Emden goose (in vitro only) eggs were respectively purchased from the White Leghorn chicken flock of the Canadian Food Inspection Agency (Ottawa, ON, Canada), King Cole Ducks Ltd. (Whitchurch-Stouffville, ON, Canada), and a local producer (ON, Canada). Chicken eggs were specific pathogen free (SPF). Eggs were incubated at 37.5 °C at 60–75% humidity and rotated every 2 h using a commercial incubator (GQF Manufacturing, Savannah, GA, USA).

Finite chicken, duck, and goose embryo fibroblasts (CEF, DEF, and GEF, respectively) were produced from embryos, using standard protocols [16]. Briefly, at 11 days of embryonation (DOE) for chicken, and 13 DOE for duck and goose, eggs were decontaminated by submersion in 70% ethanol and the trunk of harvested embryos was minced and dissociated with 0.25% trypsin (HyClone, London, ON, Canada). The supernatant was then filtered through a 40-μm cell strainer (Corning, Corning, NY, USA), and fetal bovine serum (FBS; Wisent, QC, Canada) was added to a final concentration of 5% (*v*/*v*) to inactivate the trypsin. Cells were seeded onto T-175 flasks (Fisher, Mississauga, ON, Canada) and routinely passaged in maintenance media (Dulbecco’s modified Eagle’s medium (DMEM; Wisent) with 10% FBS and 1% penicillin/streptomycin/amphotericin (PSA; HyClone)) at 37 °C with 10% CO_2_. All cell stocks were tested for presence of ABBV-1 by RT-qPCR at the Animal Health Laboratory (AHL), the provincial laboratory for animal health testing in Ontario.

### 2.2. Production of Virus Stock and Titration

ABBV-1 was harvested from persistently infected GEF (ABBV-GEF) using a freeze-thaw method [9]. Cells grown in T-175 flasks with maintenance media were supplemented with 10 mM sodium butyrate (Fisher) for 48 h. Media was then removed and replaced with 10 mL of sterile double distilled water for 2 h at room temperature before freezing at −80 °C. After thawing, the remaining cells were scraped off the surface of the flask and virus-containing supernatant was transferred to a 50 mL centrifuge tube (Fisher) and vortexed for 30 s before being centrifuged at 2000× *g* for 5 min to pellet cellular debris. The virus-containing supernatant was moved to a clean centrifuge tube and incubated overnight at 4 °C with shaking in a 1:2 ratio of polyethylene glycol 8000 (PEG8000; Fisher) with 0.4 M sodium chloride (Fisher) to supernatant. Virus was pelleted by centrifugation at 3500× *g* for 15 min at 4 °C, and the pellet was re-suspended in phosphate buffered saline (PBS; Fisher) with 1% FBS, with a final ratio of 1:500.

Virus was titrated using 96-well plates and a tissue culture infectious dose 50% (TCID_50_) assay in DEF, and immunofluorescence (IF; see below) was used for virus detection as previously described [9]. The TCID_50_ titre was calculated according to the Karber method, and converted to focus-forming units (FFU) by multiplying by 0.69 [17]. Virus stock was stored in aliquots at −80 °C.

### 2.3. Immunofluorescence

Immunofluorescence was done as previously described [9]. Briefly, cells were fixed and permeabilized with ice-cold methanol-acetone (1:1 ratio; Fisher) at −20 °C for 20 min, blocked for 1 h at room temperature in 5% normal goat serum (Sigma, St. Louis, MO, USA), and incubated overnight at 4 °C with a rabbit monospecific antibody against the ABBV-1 N protein (Pacific Immunology, Ramona, CA, USA), used at 1 μg/mL. Reaction was visualized using a goat-anti-rabbit secondary antibody conjugated with AlexaFluor488 (Thermo Fisher, Mississauga, ON, Canada) used at 1 μg/mL, with a DAPI counterstain (Fisher).

### 2.4. ABBV-1 Growth Curves

#### 2.4.1. Multi-Step Growth Curves

An experimental outline of the multi-step growth curve can be found in Supplemental Material Figure S1. A total of 0.1 × 106 CEF, GEF, and DEF were each plated into separate wells (*n* = 3 for each species) of a 12-well plate (Fisher), and let to adhere overnight. The following day, cells were infected with ABBV-1 at a multiplicity of infection (MOI) of 0.01 with virus let to adsorb for 1 h before the inoculum was removed, at which point cells were rinsed 3 times with PBS and replenished with fresh maintenance media. One additional well per species was treated with an equivalent volume of PBS as a negative control. Cells were first passaged 2 days post-infection (dpi) and then every other day for the remaining course of the experiment. A 1:3 ratio was used for each passage, with 2/3 seeded into one well of a 6-well plate (Fisher) and the remaining 1/3 into one well of a 24-well plate (Fisher) to be tested by IF for ABBV-1 the following day, to determine the percentage of infected cells over time (see below). Overall, IF was conducted over 10 passages, corresponding to 3, 5, 7, 9, 11, 13, 15, 17, 19, and 21 dpi. At 21 dpi, remaining cells in each well were detached with trypsin, counted (Countess II Automated Cell Counter (Fisher)), and lysed by 3 cycles of freeze/thaw to release cell-associated virus. Clarified supernatant was titrated for presence of ABBV-1 by TCID50 as described above.

#### 2.4.2. Single-Step Growth Curves

An experimental outline of the single-step growth curve can be found in Supplemental Material Figure S2. A total of 0.05 × 10^6^ CEF, GEF, and DEF were plated in separate wells of a 24-well plate (*n* = 15 wells for each species) and let to adhere overnight. The following day, cells were infected with ABBV-1 at an MOI of 5 with virus let to adsorb for 1 h before inoculum was removed, at which point cells were then rinsed 3 times with PBS, and replenished with fresh maintenance media. Five additional wells per species were treated with an equivalent volume of PBS as a negative control. At 0, 1, 2, and 4 dpi, IF was used to detect the percentage of infected cells at each time point (see below). Cells in the remaining four wells per species (3 infected and one control) were split in a 1:3 ratio as described above, and IF for virus was carried out at 6, 10, 14, and 21 dpi Supplemental Material Figure S2. At 21 dpi, remaining cells in each well were detached with trypsin, counted (Countess II Automated Cell Counter (Fisher)), and lysed by 3 cycles of freeze/thaw to release cell-associated virus. Clarified supernatant was titrated for presence of ABBV-1 by TCID_50_ as described above.

#### 2.4.3. Determining Percentage of Infected Cells

The number of ABBV-1 reactive cells was plotted over time to assess virus growth. For assessment of percentage of fluorescent cells, three pictures were taken per well at a 40× magnification (ZEISS Axio Observer.A1) using two channels (DAPI and FITC). For each picture, the total number of cells were counted using the DAPI (blue) channel, and the number of infected cells was determined by counting immunoreactive cells using the FITC channel (green). Therefore, considering three wells for each time point, the number of infected cells over the total number of cells per time point was determined by averaging the data from 9 pictures. Cell counting was assisted using ImageJ software (National Institute of Health, Bethesda, MD, USA).

### 2.5. In Ovo Inoculation and Tissue Harvest

#### 2.5.1. Experimental Design

A diagram of the experimental plan for the in ovo experiment is shown in Supplemental Material Figure S3. Eighty (*n* = 80) fertilized eggs per species were obtained and incubated as described above. Half (*n* = 40) were inoculated in the yolk sac and the other half in the chorioallantoic cavity. For each route, half of the embryos (*n* = 20) were inoculated either with ABBV-1 or sterile PBS (control). On account of different developmental rates and times at hatch (i.e., 21 days for chickens and 28 days for ducks), inoculation and embryo harvest were carried out at different days of embryonation (DOE). Yolk inoculation was conducted at 5 DOE in chickens, and 8 DOE in ducks; allantoic inoculation was conducted at 9 DOE in chickens, and 12 DOE in ducks. Therefore, considering the inoculation route and inoculum type, for each species there were four groups, each of *n* = 20 embryos (yolk/ABBV-1, yolk/PBS, chorioallantoic/ABBV-1, chorioallantoic/PBS). From each group, *n* = 10 embryos were harvested at two time points: early and late embryonation. Early harvest was 11 DOE for chickens, 15 DOE for ducks; late harvest was 19 DOE for chickens, 24 DOE for ducks. Half of the embryos (*n* = 5) sampled at each time point were used for histology/immunohistochemistry (IHC), and half for RNA extraction.

#### 2.5.2. Inoculation and Tissue Harvest

Inoculation into the yolk and chorioallantoic sacs was conducted according to standard procedures and sterile techniques [18], using 10^5^ FFU of ABBV-1 in 0.1 mL of PBS with 1% FBS, or an equivalent volume of PBS. After inoculation, eggs were candled every day to assess embryo viability. Embryos that died the day after inoculation were discarded (death interpreted as a consequence of inoculation); embryos that died before harvesting time were regularly sampled as described below.

At the set time points described above, embryos were euthanized by refrigeration from 6 h to overnight prior to harvest; and the embryos were transferred into a sterile petri dish for dissection. Harvested tissues for RNA extraction and histology included vitelline membrane with yolk, brain, spinal cord, liver, proventriculus and ventriculus (P/V). Allantoic fluid was harvested for RNA extraction, and chorioallantoic membrane for histology. For RNA extraction, fluids and tissues were stored in screw cap tubes and frozen at −80 °C. For histology, tissues were fixed in 10% neutral buffered formalin for 48 h and then preserved in 70% ethanol until being embedded in paraffin wax and routinely processed for hematoxylin and eosin (HE) staining and IHC.

### 2.6. Histology and Immunohistochemistry

Histology was carried out on all sampled tissues for evaluation of lesions. IHC was only done on brain and spinal cord tissue from *n* = 2 controls and *n* = 3 ABBV-1 inoculated embryos per harvest time point. Nervous tissue was considered the mostly likely site of virus replication based on literature sources [11]. IHC was conducted as reported previously [19]. Briefly, after deparaffinization, sections were unmasked using Proteinase K and blocked according to the manufacturer’s instructions (Agilent Technologies, Mississauga, ON, Canada). The primary antibody was the same used for IF, and was applied onto the slides for 30 min at room temperature at 1:6000 dilution, followed by a polymer (Envision, Agilent Technologies) conjugated with peroxidase. Positive reaction was visualized using NovaRED chromogen (Vector Laboratories, Burlington, ON, Canada). Positive control sections derived from the brain of Canada geese (*Branta canadensis*) naturally infected with ABBV-1 [1].

### 2.7. RNA Extraction and RT-qPCR

RNA was extracted from 300 µL of allantoic fluid or yolk, or 300 mg of solid tissue (brain, liver, P/V) using TRIzol/chloroform after homogenization in a tissue homogenizer (Precellys; ESBE Scientific, Markham, ON, Canada) with 1.0 mm sterile glass beads. RNA was then purified using RNeasy Mini Kit (Qiagen, Toronto, ON, Canada). Viral RNA was detected from purified RNA using the Luna^®^ Universal One-Step RT-qPCR kit (New England BioLabs, Whitby, ON, Canada), as previously described [9]. Forward (5′-ATGCACTTGCACTCTTAGAC-3′) and reverse (5′-TCCCCATAAAACCTCCCAAC-3′) primers were designed to target the ABBV-1 N gene along with a probe (5′-6FAM-CCCTGCCCGCAGAGAGAAATTCCAT-BHQ-3′) for detection. Reactions were prepared according to manufacturer instructions, with 100 ng of RNA template.

Viral RNA in tissues was quantitated using a standard curve, which was developed as follows. Virus was harvested from 1.0 × 10^6^ ABBV-GEFs by 3 cycles of freeze/thaw, and released virus titrated by IF as described above. RNA was extracted from an equivalent number of cells using RNeasy Mini Kit (Qiagen), and 10-fold serial dilutions of RNA were used as template for RT-qPCR, using the same conditions previously described. The threshold cycle (i.e., Ct value) of each dilution was then plotted against the relative amount of FFU, to create a standard curve. Since total RNA was extracted from the whole amount of harvested samples (either 300 mg or 300 µL), the standard curve was further modified to plot Ct value versus FFU/mg or µL of sample. Samples were considered positive with Ct values ≤30.

### 2.8. Statistical Analysis

For the growth curves, differences between the means of the proportions of infected cells at each time point between species were assessed using a two-way analysis of variance (ANOVA), wherein the variables included species (i.e., type of cell) and time point, followed by a post-hoc Tukey’s test for multiple comparisons. To assess differences in virus titres between GEF, DEF, and CEF at the end of the growth curves, means of viral titres per one million cells were log_10_ transformed and tested by a two-tailed *t*-test.

For the in ovo experiment, differences between the proportion of positive/negative samples between species were determined using a Chi-squared test. All statistical analyses were conducted using GraphPad Prism 8.0 software (GraphPad, La Jolla, CA, USA).

## 3. Results

### 3.1. ABBV-1 Can Efficiently Spread in GEF and DEF, but Not CEF

**Figure 1:**
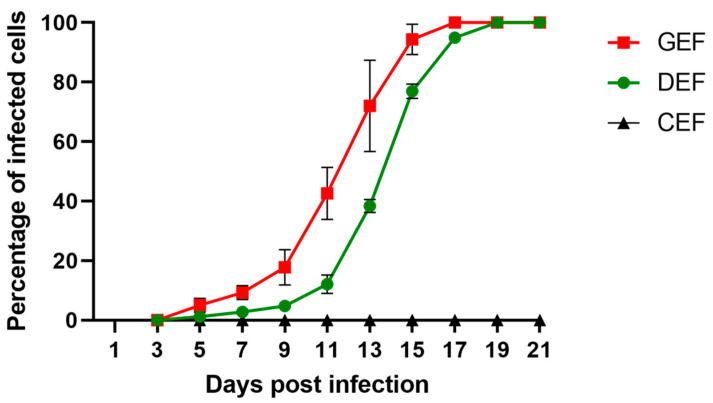
Multi-step growth curves of aquatic bird bornavirus 1 (ABBV-1) in primary embryonic fibroblasts from chicken (CEF), duck (DEF), and goose (GEF). Cells were infected at a multiplicity of infection (MOI) of 0.01, and at 3, 5, 9, 11, 13, 15, 17, 19, and 21 days post infection (dpi) a portion of infected cells was fixed and virus was detected using immunofluorescence. Cells were counterstained with DAPI to determine percentage of infected cells over the total. Each data point represents mean +/− standard deviation (*n* = 3).

GEF, DEF, and CEF were infected with ABBV-1 at an MOI of 0.01, split every other day, and the proportion of infected cells at each time point was determined by IF. Dissemination of ABBV-1 in GEF and DEF was slow initially, with less than 20% of cells positive for the N protein within the first 11 days; however, there was a significant difference in the proportion of positive GEF compared to DEF by 9 dpi (*p* < 0.0001) (Figure 1). Exponential increase in the proportion of infected GEF and DEF was seen between 11 and 17 dpi, and virtually 100% of cells were immunoreactive after 17 dpi for GEF, and 19 dpi for DEF. The rate of growth was faster in GEF than in DEF, with the proportion of positive cells significantly higher in GEF from 9 dpi until 15 dpi, compared to DEF (*p* < 0.0001 for each time point in between). In contrast, CEF showed no immunofluorescence signal over the 21-day period. Negative controls showed no immunoreactivity.

### 3.2. GEF, DEF, and CEF Can Be Infected with Large Doses of ABBV-1; However, a Persistent Infection Cannot Be Established in CEF

**Figure 2:**
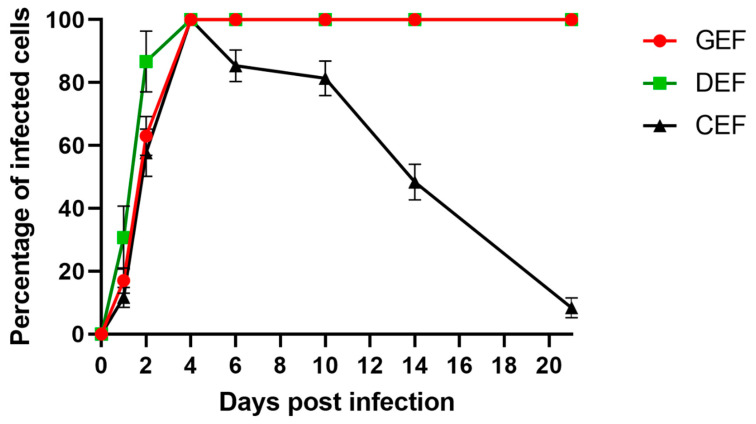
Single-step growth curves of ABBV-1 in primary embryonic fibroblasts from chicken (CEF), duck (DEF), and goose (GEF). Cells were infected at a multiplicity of infection (MOI) of 5, and at 1, 2, 4, 6, 10, 14, and 21 days post infection (dpi) a population of cells were fixed and virus was detected using immunofluorescence. Cells were counterstained with DAPI to determine percentage of infected cells over time. Each data point represents mean +/− standard deviation (*n* = 3).

GEF, DEF, and CEF were infected with ABBV-1 at an MOI of 5, and the proportion of infected cells was evaluated by IF over a three-week period. Virus infection was detected rapidly in GEF, DEF, and CEF, with 20% of cells immunoreactive within 1 dpi (Figure 2). By 4 dpi, virtually 100% of GEF, DEF, and CEF were infected. Starting at 6 dpi, the proportion of infected CEF significantly decreased compared to GEF and DEF (*p* < 0.0001), declining to 90% at 6 dpi, and dipping to <10% by the end of the experiment (21 dpi). The percentage of infected cells of GEF and DEF cultures did not fluctuate after 100% infection was achieved at 4 dpi, and was maintained through the entire length of the experiment (Figure 2).

### 3.3. GEF and DEF, but Not CEF, Can Produce Infectious ABBV-1

At the end of each growth curve, the number of cells in the remaining wells were counted, and the virus was released from the cells by freeze/thaw. The titre, expressed as FFU/1.0 *×* 10^6^ cells, was averaged between the three replicates for each species and FFU were log_10_ transformed. GEF and DEF produced on average 10^3.94^ and 10^4.07^ FFU/1.0 *×* 10^6^ cells, respectively, at the end of the multi-step growth curve, and 10^3.77^ and 10^3.85^ FFU/1.0 *×* 10^6^ cells at the end of the single-step growth curve. The magnitude of virus titres produced in GEF and DEF was not significantly different (*p* > 0.05). No detectable amounts of infectious virus were produced from CEF from either multi- or single-step growth curves (Table 1).

### 3.4. Immunofluorescence Signal is Nuclear and Cytoplasmic in GEF and DEF, but Only Nuclear in CEF

**Figure 3:**
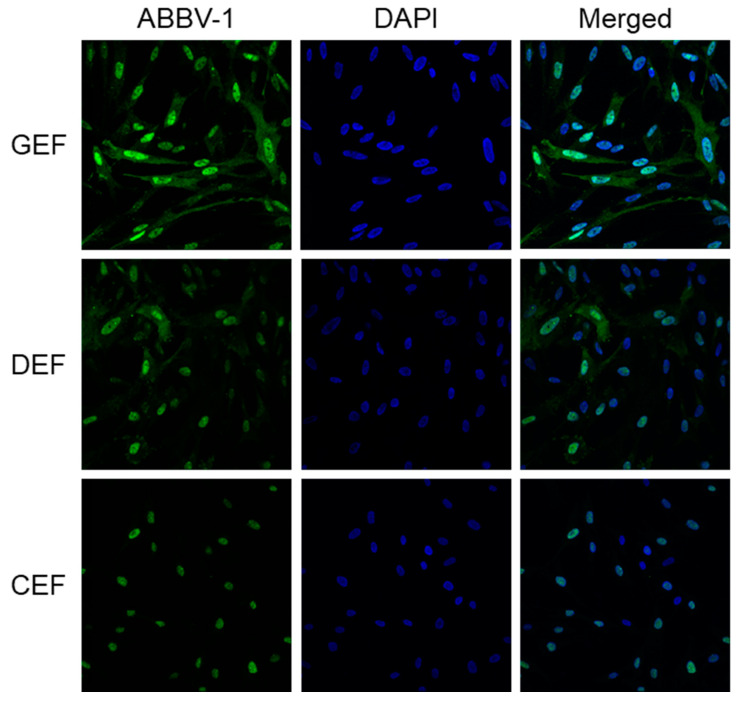
Patterns of immunofluorescence reactivity in embryonic fibroblasts infected with ABBV-1. Embryonic fibroblasts from chicken (CEF), duck (DEF), and goose (GEF) were infected with ABBV-1 at a multiplicity of infection (MOI) = 5. Immunofluorescence for ABBV-1 antigen was conducted at 6 days post infection (dpi). Column of panels on the left shows ABBV-1 immunofluorescence; column of panels in the middle shows DAPI counterstain alone; and column of panels on the right shows the merged images. Each row of panels indicates a different species. Magnification, 63× for all panels.

The immunofluorescence reactivity pattern in GEF and DEF was characterized by speckled nuclear and diffuse cytoplasmic immunoreactivity (Figure 3). Reactivity in CEF displayed predominately nuclear staining, with minimal to no cytoplasmic reactivity (Figure 3). Immunoreactivity pattern remained the same throughout the course of the growth curve experiments in all three species.

### 3.5. In Ovo Inoculation, Egg Incubation, and Tissue Harvest

Fertilized chicken and duck eggs were inoculated into the yolk sac or the allantoic cavity with 10^5^ FFU of ABBV-1 or an equivalent volume of PBS. Embryos were harvested at early and late embryonation time for tissue collection. Four chicken and duck eggs (total, 8) were either not fertilized or contained dead embryos prior to inoculation and were discarded, leaving a total of 76 eggs for inoculation. Within 24 h after inoculation, 16 embryos across both species died; this was assumed a consequence of inoculation, as this number includes both ABBV-1 and PBS inoculated eggs. As a result, the final number of chicken and duck embryos that were used in the experiment were respectively 67 and 69, for a total of 136 embryos.

Due to the decreased volume of the chorioallantoic sac at the later stages of embryo development, 15 allantoic fluid samples could not be harvested from duck eggs for RNA extraction. Thus, 178 and 173 tissues from ABBV-1- and PBS-inoculated embryos, respectively, were available for RNA extraction. A total of 192 tissues from ABBV-1 and PBS-inoculated embryos were available for histopathology, and 40 were tested by IHC (brain and spinal cord only).

### 3.6. ABBV-1 Does Not Replicate or Cause Lesions in Embryonic Tissues

RT-qPCR of brain, P/V, liver, yolk and allantoic fluid was negative for all tested samples with two exceptions: the brain from a yolk-inoculated chicken at early harvest (Ct = 29.74), and the yolk of a yolk-inoculated duck at late harvest (Ct = 29.09) (Table 2). These correspond to estimated titres of 1.6 × 10^−^^1^ and 1.7 × 10^−1^ FFU/mg of tissue, respectively.

**Figure 4:**
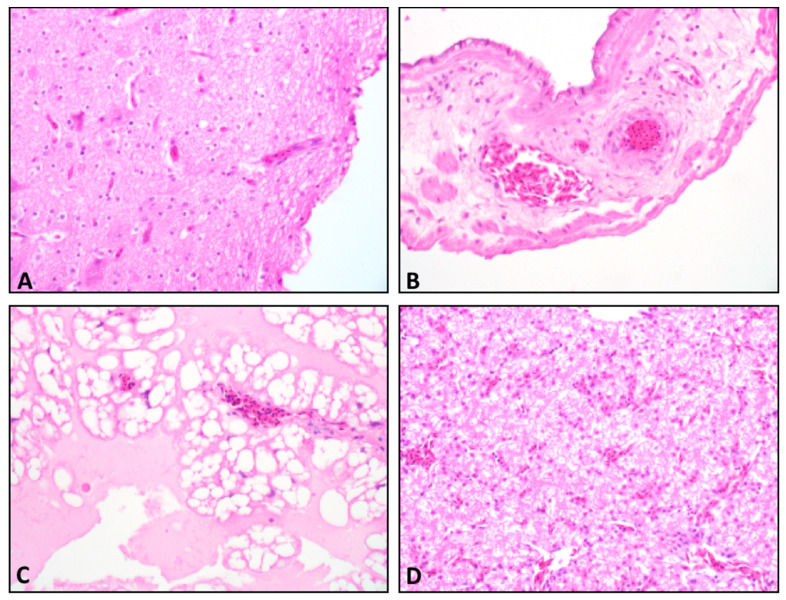
Hematoxylin and eosin (HE) stained tissue from ABBV-1 inoculated embryos. Tissue sections showing normal tissue and no lesion development; for all panels, original magnification, 40×. (**A**) Late harvest brain from a duck embryo inoculated with ABBV-1 via the yolk sac. (**B**) Late harvest chorioallantoic membrane (CAM) of a duck inoculated with ABBV-1 via the chorioallantoic cavity. (**C**) Late harvest vitelline membrane of a chicken inoculated with ABBV-1 via the yolk sac. (**D**) Late harvest liver from a duck infected with ABBV-1 via the chorioallantoic cavity.

Brain, spinal cord, liver, P/V, and chorioallantoic and vitelline membranes were assessed for presence of microscopic lesions. No inflammatory lesions consistent with ABBV-1 infection, such as lymphoplasmacytic infiltration and presence of mononuclear perivascular cuffs in the central (brain and spinal cord) or peripheral nervous tissues were observed in tissues from any ABBV- or PBS-inoculated embryos (Figure 4). Immunohistochemistry on brain tissues and spinal cords from 40 embryos showed no immunoreactive cells, regardless of inoculum, route, or harvest time [20].

## 4. Discussion

In this study, we assessed the replication kinetics of ABBV-1 in primary fibroblasts derived from chicken, duck, and goose embryos through multi- and single-step growth curves; and used an in ovo experimental infection model to determine the ability of ABBV-1 to replicate and cause lesions in chicken and duck embryos. Compared to CEF, GEF and DEF could best support ABBV-1 replication and establishment of persistent infection. Multi-step growth curves showed that ABBV-1 is not able to spread in CEF, while single-step growth curves showed that CEF could be infected, but could not sustain ABBV-1 persistent infection. In ovo inoculation showed that regardless of host, inoculation route, or harvest time, ABBV-1 fails to replicate or cause lesions in the tested embryonic tissues.

Infection of GEF and DEF with a low MOI resulted in growth kinetics characterized by an initial slow growth, followed by exponential increase of infected cells and a plateau reached by the end of experiment, indicating infection of all cells in the population. This kinetic is similar to what is described with Borna disease virus (BoDV-1), which commonly reaches a plateau in infected cells between 15 and 30 dpi in hippocampal neurons and Vero cells, respectively [21,22]. In our study, infection of CEF with a low MOI yielded no IF-positive cells over the 21-day duration of the experiment. Studies with Estrildid finch bornavirus-1 (EsBV-1) has indicated that establishment of a persistent infection, where all cells are infected, can take up to several months [23]. In the present study, all cells were primary embryo fibroblasts, and experiments were not protracted for greater numbers of passages due to cellular senescence. Although no immunoreactivity in CEF infected with ABBV-1 was observed at low MOI, virus replication may have been present at very low levels, detectable only by a more sensitive method, such as RT-qPCR. Indeed, immortalized chicken embryo fibroblasts (DF1) had detectable levels of ABBV-1 RNA upon infection without any immunoreactivity noticed by IF [9].

In single-step curves, ABBV-1 replicated in GEF and DEF with a trend similar to what seen in multi-step growth curves, albeit at a much faster rate, and were able to maintain an infected state for three weeks (i.e., persistent infection). Similar to GEF and DEF, CEF also rapidly became infected upon inoculation with ABBV-1 at a high MOI, however the intensity of fluorescence signal and amount of positive cells decreased with the number of passages. The initial burst of replication is likely due to infection with an overwhelming amount of virus, followed by decreasing replication rates, possibly caused by poor adaptation of ABBV-1 to the chicken host.

Differences in the staining pattern between CEF and the other cells were seen by immunofluorescence. CEF showed both nuclear and cytoplasmic reactivity initially, which at later time points became predominately nuclear, while in the other cells signal appeared to be both cytoplasmic and nuclear. In BoDV-1, the nucleoprotein has been shown to exist as two different isomers: p40, which is nuclear, and p38, which is cytoplasmic and transcribed without the N-terminal nuclear localization signal [24]. Both isoforms are critical for nucleo-cytoplasmic shuttling of viral RNA, and are essential for BoDV-1 replication [25,26]. The lack of cytoplasmic staining seen in CEF at later time points may indicate inefficient replication. There was no measurable infectious virus produced by infected CEF in spite of the presence of immunoreactive nuclei. Absent cytoplasmic reactivity may be due to impaired nucleocytoplasmic shuttling of the nucleoprotein, although the two isoforms have yet to be shown to exist in avian bornaviruses.

The differential growth of ABBV-1 in the cell cultures of the present work are likely to be explained by species-specific differences in the innate immune response, or permissivity to virus replication. Future research will need to address the differences in expression of antiviral genes that are activated upon ABBV-1 infection. Additionally, the use of neural cell lines might provide a more realistic understanding of replication kinetics, however the use of the fibroblast model to address the question of host range appears appropriate, considering that goose and duck fibroblasts were able to be infected and establish a persistent infection.

In order to better answer ABBV-1 host restriction, we also attempted infection of embryonated eggs. In ovo models are used frequently in virology for both virus propagation and pathogenesis studies. Inoculation of embryonated eggs into the allantoic cavity is a common method for growing influenza virus and Newcastle disease virus (NDV), where the virus infects the chorioallantoic membrane (CAM), replicates, and is released back into the allantoic fluid [27,28]. In cases with mesogenic and velogenic NDV, the virus is capable of infiltrating through the outermost epithelial layer of CAM to access the blood, and become systemically distributed throughout embryonic tissues [29]. Yolk sac inoculation is less common, but has been utilized as a route to isolate and grow Marek’s disease virus (*Gallid alphaherpesvirus 2*; *GaHV-2*) and Coxsackie virus [30,31]. In these cases, the virus infiltrates the yolk sac membrane before distributing throughout the embryo. In the present study, there were only two samples that tested positive for viral RNA by RT-qPCR: a yolk-inoculated early harvest chicken brain, and a yolk-inoculated late harvest duck yolk. Both samples had Ct values barely above the cut-off threshold, and had estimated viral titres less than 1 FFU/mg of tissue. ABBV-1 may not be able to infiltrate the vitelline or chorioallantoic membrane to get to a permissive cell or tissue (i.e., neurons, nervous tissue) and subsequently degraded, resulting in the majority of samples testing negative. The two samples that were positive had low estimated titres, suggesting that these likely represent remnants of the initial inoculum.

In addition to RT-qPCR, virus replication was evaluated by IHC using a monospecific antibody against the ABBV-1 N protein, in both the brain and spinal cord of inoculated embryos. In naturally infected Canada geese, brains show scattered immunoreactivity for ABBV-1 in the nuclei of both neurons and glial cells [1]. In the present study, immunoreactivity was not observed in any samples. This corroborates the finding from the RT-qPCR results, and the claim that the two weak positives likely represent a low-level contamination.

Microscopic lesion development was assessed in multiple embryonic tissues. The two most common lesions documented with avian bornavirus infections in multiple avian species (including ABBV-1 infection in waterfowl) include lymphoplasmacytic infiltration of the peripheral ganglia and perivascular cuffs of mononuclear cells in the CNS [1]. No lesions were observed in the central and peripheral nervous tissue, as well as other organs, in any of the tissues from ABBV-1- or mock-inoculated embryos. As bornaviruses do not cause direct cell lysis, lesion development is believed to be a consequence of inflammation mediated by the cell-mediated immune response of the host [10]. The lack of a fully-functioning immune system in the developing embryo [32] could explain the absence of lesions observed in the embryonic tissues of this study. Overall, lack of lesions and the negative IHC and RT-qPCR results, suggest that ABBV-1 did not spread or replicate in embryonic tissues, and did not elicit lesion development.

For a cell-associated virus, the nervous tissue (i.e., the site of ABBV-1 replication) might have been inaccessible from a distant site of inoculation (i.e., chorioallantoic and vitelline sac). Additionally, the incubation time before hatch might have been too short for ABBV-1 to fully replicate in tissues. In fact, experimental inoculation of cockatiels with brain homogenate from an African gray parrot infected with PaBV-4 failed to show clinical signs until 21 days after infection, and, in another study, cockatiels inoculated with PaBV-4 did not develop clinical signs for 110 days [33,34].

## 5. Conclusions

Overall, the ability of ABBV-1 to propagate faster and at higher titres in primary embryonic fibroblasts from waterfowl (goose and duck) compared to chicken suggests that these hosts have different levels of permissivity to infection, which may reflect the high prevalence of ABBV-1 in wild anatids, and the relative lack of reported natural infection in gallinaceous species. To further characterize host restriction in a more relevant experimental system, we also endeavoured to model infection in chicken and duck embryos. Unfortunately, an in ovo system appears to be inadequate to assess host restriction, tissue tropism, or pathogenesis of ABBV-1, as no viral RNA, antigen, or lesions could be detected in the embryos at any time point after ABBV-1 inoculation. The inability of ABBV-1 to infect embryos suggests that vertical transmission may not be an important route of ABBV-1 dissemination, even for susceptible species such as waterfowl. As the in ovo system did not successfully model ABBV-1 infection, in vivo trials are the necessary next step to fully understand the pathogenesis and host restriction of ABBV-1, especially for domestic waterfowl, which appear to be highly permissive to ABBV-1 infection at a cellular level.

## Figures and Tables

**Table 1:** Production of infectious ABBV-1 in primary embryonic fibroblasts of chicken (CEF), duck (DEF), and goose (GEF), at the end of multi- and single-step growth curves (21 days post-infection). Each value represents the log_10_ of the mean titre (expressed in FFU) +/− standard deviation per 10^6^ cells, as assessed in *n* = 3 replicates. Values were not given for CEF, as no infectious virus could be detected by immunofluorescence in these cells. N/A = no titre available for CEF; ns = not significant.

	GEF	DEF	CEF	Statistically Significant
Multi-Step	3.94 ± 0.06	4.07 ± 0.24	N/A	ns
Single-Step	3.77 ± 0.11	3.85 ± 0.21	N/A	ns

**Table 2:** Summary of RT-qPCR results from tissues of chicken and ducks embryos inoculated with ABBV-1. Each value represents the number of positive tissues over the total tested. AF: allantoic fluid; PV: proventriculus and ventriculus.

	Chicken					Duck				
Inoculation Route/Harvest Time	Brain	PV	Liver	Yolk	AF	Brain	PV	Liver	Yolk	AF
Yolk/Early	1/5	0/4	0/4	0/4	0/4	0/5	0/4	0/4	0/5	0/5
Allantoic/Early	0/5	0/5	0/5	0/5	0/5	0/5	0/5	0/5	0/5	0/5
Yolk/Late	0/4	0/4	0/4	0/4	0/4	0/4	0/4	0/4	1/4	0/2
Allantoic/Late	0/5	0/5	0/5	0/5	0/5	0/5	0/5	0/5	0/5	N/A
Subtotals	1/19	0/18	0/18	0/17	0/19	0/19	0/19	0/18	1/19	0/12

## Supplementary Materials

The following are available online at https://www.mdpi.com/1999-4915/12/11/1272/s1, Figure S1: Experimental outline of the multi-step growth curve, Figure S2: Experimental outline of the single-step growth curve, Figure S3: Experimental outline of the in ovo study.
