# Supplementary material for: In Vitro and In Ovo Host Restriction of Aquatic Bird Bornavirus 1 in Different Avian Hosts
Source: Viruses. 2020 Nov 7;12(11):1272. doi: 10.3390/v12111272 (PMC7694974; doi:10.3390/v12111272)
Supplement: Supplementary file 1 [file viruses-12-01272-s001.zip › supplementary-figures-submission.pdf]

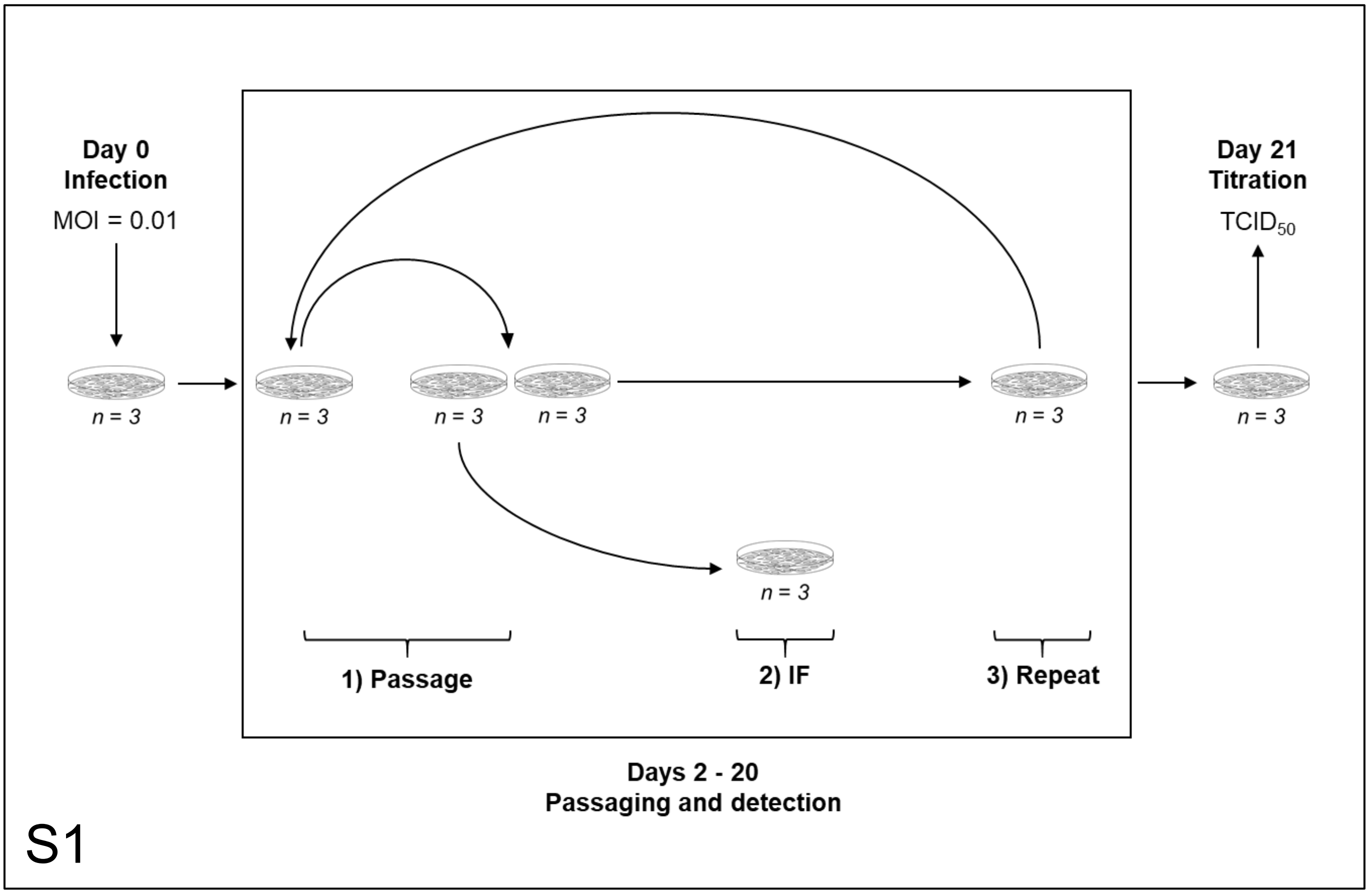

Figure S1. Experimental outline for the multi-step growth curves.

Primary embryonic fibroblasts of chicken (CEF), duck (DEF) and goose (GEF) were infected with an MOI of 0.01 in triplicate. Two days post-infection (dpi) cells were passaged at a 1:3 ratio, with 1/3 plated for immunofluorescence (IF) the following day, and the remaining 2/3 plated for continued passaging. Overall, cells were passaged every other day starting at 2 dpi until 20 dpi, with IF being performed the subsequent day following each passage. Thus, IF was performed on days 3, 5, 7, 9, 11, 13, 15, 17, 19, and 21 dpi. At 21 dpi, remaining cells were frozen and thawed, and virus was titrated by limiting dilutions ($TCID_{50}$).

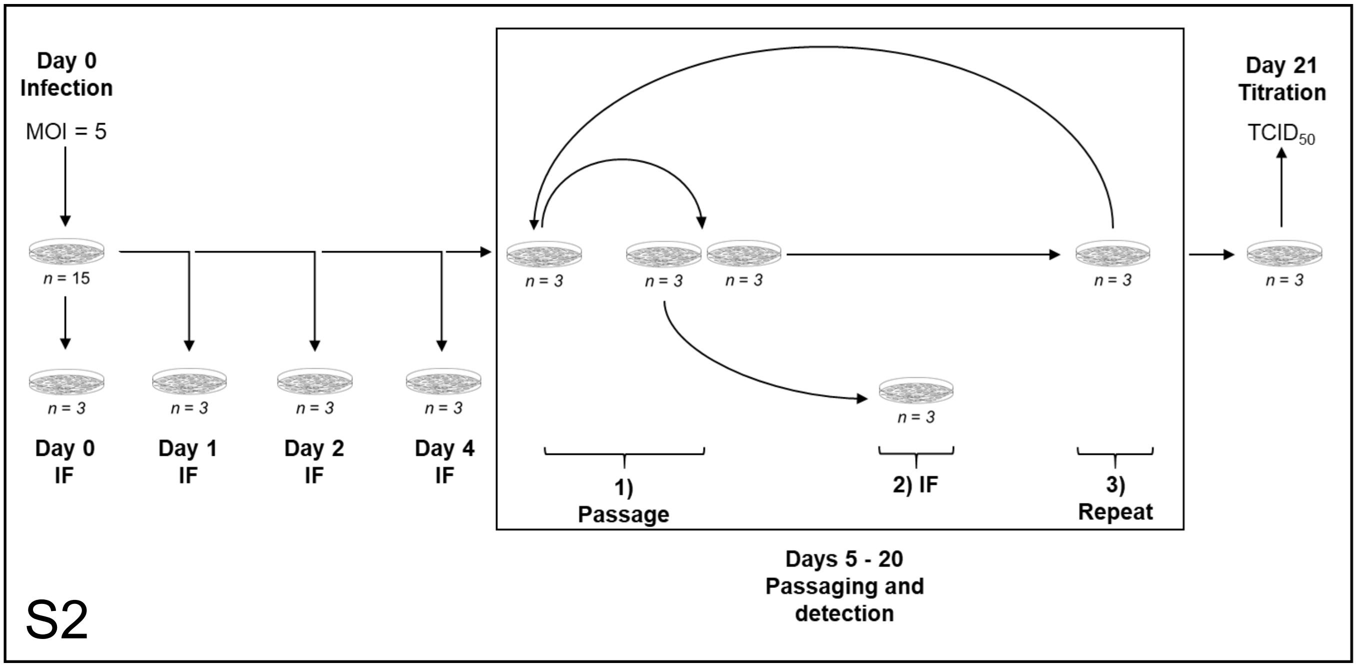

Figure S2. Experimental outline for the single-step growth curves.

Primary embryonic fibroblasts of chicken (CEF), duck (DEF) and goose (GEF) were infected with an MOI of 5, with 15 wells per species. At 0, 1, 2, and 4 days post-infection (dpi), three replicate wells from each species were fixed, and ABBV-1 was detected using immunofluorescence. The remaining three wells for each species were passaged as described for the multi-step growth curve. Overall, IF was performed on 0, 1, 2, 4, 6, 10, 14, and 21 dpi. At 21 dpi, remaining cells were frozen and thawed, and virus was titrated by limiting dilutions ($TCID_{50}$).

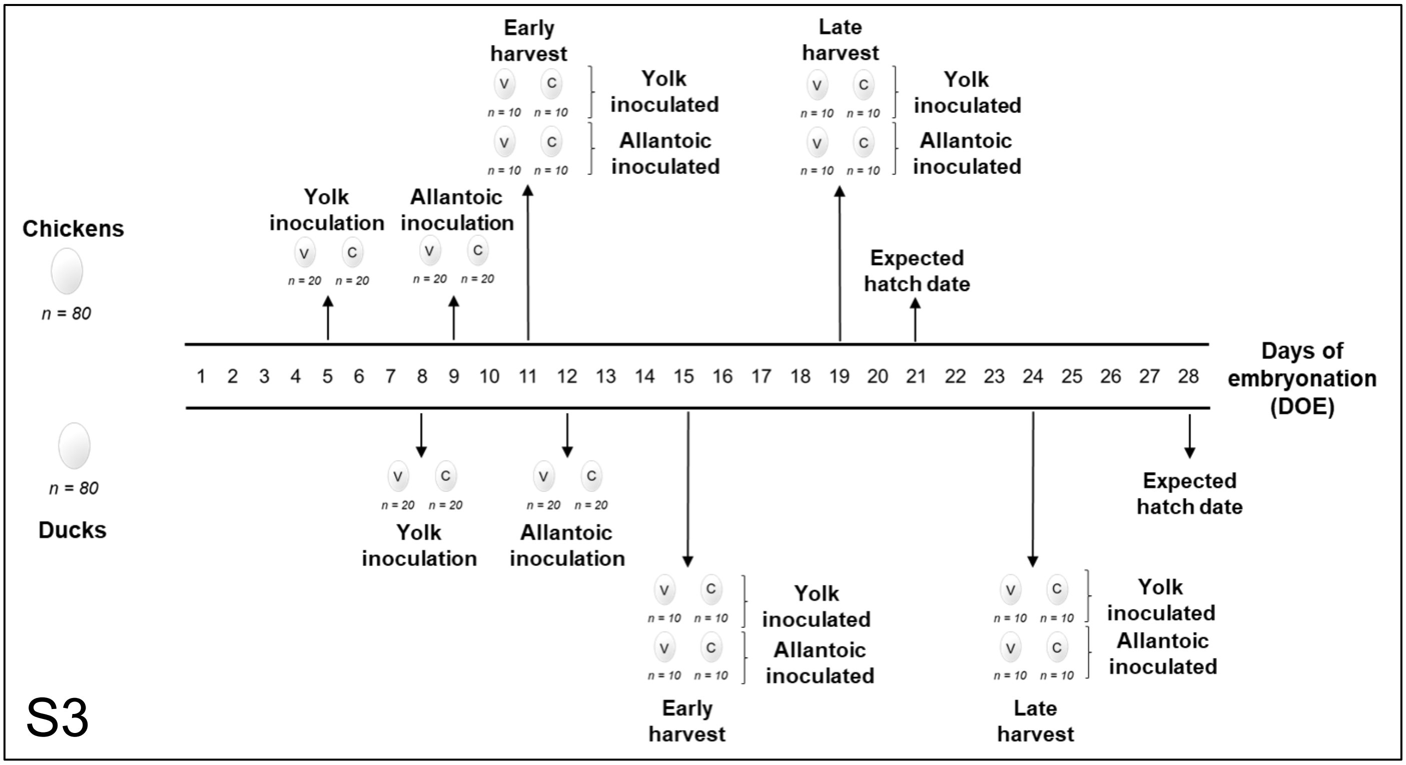

Figure S3. Experimental outline for the *in ovo* experiment.

A total of 80 fertilized chicken and duck eggs were obtained. Half (*n* = *40*) of the eggs were inoculated in the yolk sac at 5 days of embryonation (DOE) for chickens and 8 DOE for ducks. The remaining half (*n* = *40*) were inoculated into the chorio-allantoic cavity at 9 DOE for chickens and 12 DOE for ducks. At each of these inoculation times, half (*n* = *20*) were inoculated with ABBV-1 (V), and half (*n* = *20*) were inoculated with PBS as a control (C). Embryos were harvested at two time points: early, 11 DOE for chickens and 15 DOE for ducks; and late, 19 DOE for chickens and 24 DOE for ducks. Forty embryos were harvested at each harvest time, with *n* = *10* representing one of the two inoculum routes and types (i.e., *n* = 10 yolk/ABBV-1, *n* = 10 yolk/PBS, *n* = 10 chorio-allantoic/ABBV-1; *n* = 10 chorio-allantoic/PBS).
